# The zygomatic implant perforated (ZIP) flap: a new technique for combined surgical reconstruction and rapid fixed dental rehabilitation following low-level maxillectomy

**DOI:** 10.1186/s40729-017-0100-8

**Published:** 2017-07-29

**Authors:** C. J. Butterworth, S. N. Rogers

**Affiliations:** Department of Oral & Maxillofacial Surgery, University Hospital Aintree, Lower Lane, Liverpool, L9 7AL UK

**Keywords:** Low-level maxillectomy, Zygomatic implants, Zygomatic oncology implant, Fixed dental prosthesis, ZIP flap, Micro-vascular reconstruction, Radiotherapy, Early implant loading, Oral cancer rehabilitation

## Abstract

This aim of this report is to describe the development and evolution of a new surgical technique for the immediate surgical reconstruction and rapid post-operative prosthodontic rehabilitation with a fixed dental prosthesis following low-level maxillectomy for malignant disease.

The technique involves the use of a zygomatic oncology implant perforated micro-vascular soft tissue flap (ZIP flap) for the primary management of maxillary malignancy with surgical closure of the resultant maxillary defect and the installation of osseointegrated support for a zygomatic implant-supported maxillary fixed dental prosthesis.

The use of this technique facilitates extremely rapid oral and dental rehabilitation within a few weeks of resective surgery, providing rapid return to function and restoring appearance following low-level maxillary resection, even in cases where radiotherapy is required as an adjuvant treatment post-operatively. The ZIP flap technique has been adopted as a standard procedure in the unit for the management of low-level maxillary malignancy, and this report provides a detailed step-by-step approach to treatment and discusses modifications developed over the treatment of an initial cohort of patients.

## Background

The surgical management and prosthodontic rehabilitation of the maxillectomy patient is complex with a variety of options available to the head and neck cancer team ranging from simple prosthodontic obturation [1] to reconstruction using pre-fabricated or digitally planned composite flaps [2] with or without the placement of osseointegrated implants [3]. The primary aims of treatment include effective eradication of the primary tumour, closure of the resulting maxillary defect, preservation of facial form, and ideally, the restoration of the resected maxillary dentition. Whilst the techniques for surgical closure of the low-level maxillectomy defect are well established, it can be challenging to subsequently achieve effective dental rehabilitation. The use of an obturator is not without its difficulties in terms of fit, retention and comfort, as well as preventing the transgress of fluid from the mouth to the nose. Providing and maintaining an effective obturator is demanding on both the patient and prosthodontist. Although some patients are able to tolerate the use of a removable denture following treatment, depending on retention, many are unable due to the change in the oral anatomy, oral dryness and the fragility of the irradiated tissues. Sealing the defect and providing bone and soft tissue through the use of free tissue transfer has both advantages and disadvantages. Following free tissue transfer providing secondary rehabilitation might be delayed or not possible. The situation is made worse by the frequent requirement for post-operative radiotherapy, which ideally should start as soon as feasible following tumour ablation.

The development of highly specialised tools such as zygomatic, oncology and co-axis implants (Southern Implants Ltd., South Africa) have provided a platform for effective maxillary dental rehabilitation in a rapid manner following maxillary resective surgery. Boyes-Varley et al. (2007) [4] successfully demonstrated the use of early loading in this cancer setting utilising oncology zygomatic and dental implants together with prosthetic obturation. Whilst implant survival was not a problem, the amount of prosthodontic maintenance was significant and most likely related to the complex issues around establishing and maintaining an oro-nasal seal in a changing maxillectomy cavity. The technique presented here incorporates an early loading zygomatic and oncology implant protocol for maxillectomy patients together with microvascular free-flap closure of the resultant defect with a fascio-cutaneous flap and early delivery of a fixed dental prosthesis within a few weeks following surgery.

## Case presentation

**Fig. 1:**
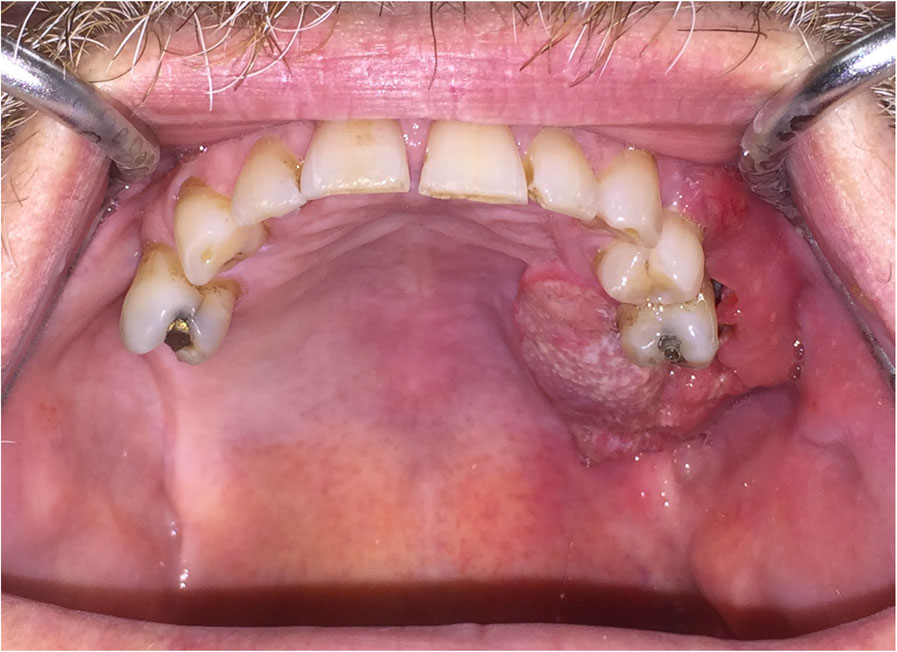
Clinical view of left-sided maxillary tumour at presentation

**Fig. 2:**
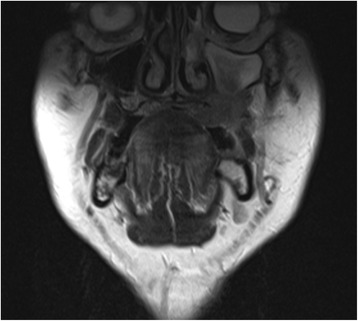
Staging MRI scan showing destructive lesion left maxilla

**Fig. 3:**
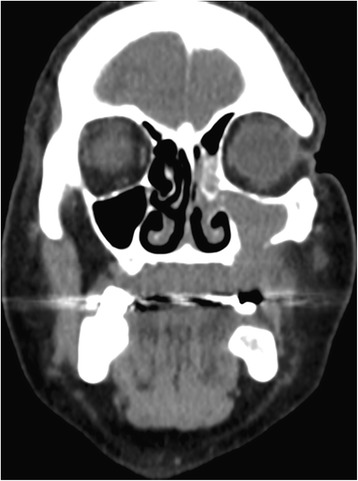
Staging CT scan confirming maxillary destruction but preservation of the orbital floor

**Fig. 4:**
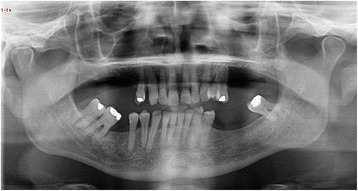
Panoramic dental radiograph showing dental status at presentation

A 66-year-old male patient presented with an enlarging mass in the left maxilla (Fig. 1). The mass had been present for a few weeks. An incisional biopsy revealed squamous cell carcinoma. Staging scans were undertaken (Fig. 2) which demonstrated a T4N0M0 maxillary alveolus tumour in close proximity to the left orbital floor with obliteration of the maxillary antrum and destruction of the lateral maxillary wall (Fig. 3). The patient was partially dentate in both jaws with no significant dental pathology (Fig. 4).

The findings were discussed with the patient together with the treatment options for this malignant tumour requiring a low-level Brown class 2b maxillectomy [5]. The patient preference was not to have prosthodontic obturation but rather reconstruction using microvascular free tissue transfer. In view of the unilateral low-level nature of the tumour, a soft tissue reconstruction combined with primary insertion of zygomatic implants to support a subsequent fixed dental prosthesis on a shortened dental arch concept was considered the best option. The remaining molar teeth were planned for extraction based on the potential need for post-operative radiotherapy and likelihood of trismus post-operatively. The remaining maxillary teeth on the non-defect right-hand side were planned for extraction to allow either the placement of immediate dental implants or the placement of conventional zygomatic implants depending on the state of the socket anatomy post-extraction.

Dental impressions were taken to allow construction of a maxillary complete denture template to both aid the placement of the zygomatic implants on the defect side and to act as an occlusal registration device during surgery. The occlusal vertical dimension was also measured between nasal tip and chin point to allow subsequent registration to occur at the correct level during surgery.

### The ZIP flap technique

**Fig. 5:**
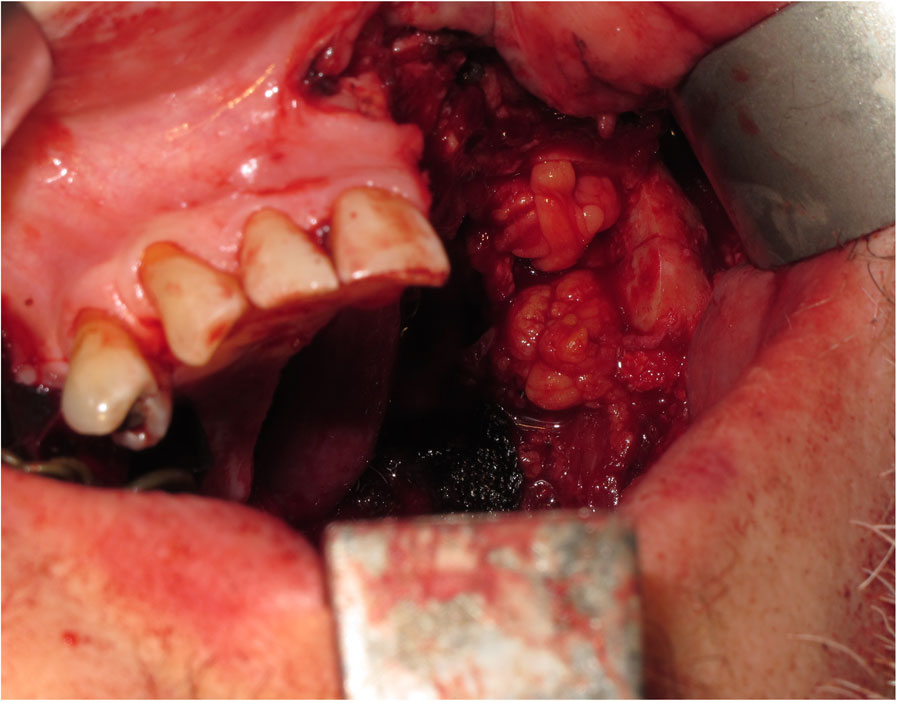
Left-sided maxillary resection (Brown class 2b)

**Fig. 6:**
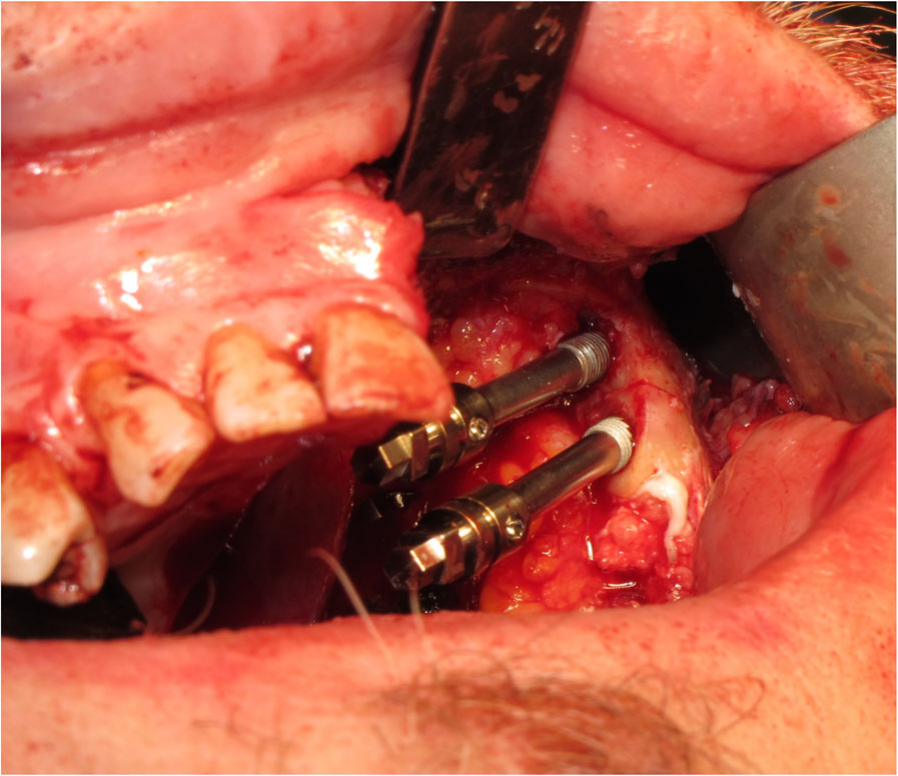
Zygomatic oncology implants sited in the residual zygomatic bone on the defect side of the maxilla

**Fig. 7:**
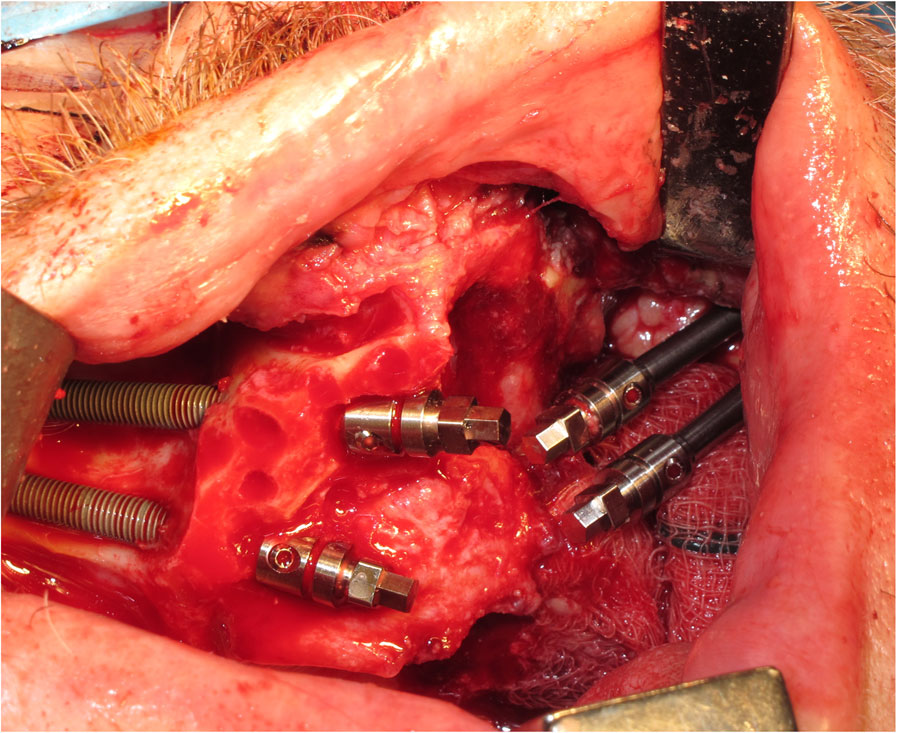
Conventional zygomatic implant insertion on the non-defect side of the maxilla following extraction of the remaining teeth and an alveoloplasty

The patient underwent tracheostomy, a limited left-sided selective neck dissection for node sampling and vessels preparation. The maxillary tumour was excised in a standard manner via an intra-oral approach with preservation of the left orbital floor (Fig. 5). The resection extended to the maxillary alveolar midline in the incisor region with extension posteriorly just into the soft palate. The defect was measured to allow the harvesting of a slightly oversized left fascio-cutaneous radial forearm flap which was carried out in parallel to the implant procedures. Following resection, the amount of bone remaining in the left zygoma was assessed and deemed satisfactory for the placement of two zygomatic oncology implants [6] (Southern Implants Ltd., South Africa) which were subsequently inserted with excellent primary stability (Fig. 6). The remaining maxillary teeth were then carefully extracted although it was not possible to preserve all the labial socket bone which was fused to several of the teeth. It was therefore decided to proceed with an alveoloplasty and insertion of two conventional zygomatic implants (Southern Implants Ltd., South Africa) on the right side which were inserted into the canine and second premolar sites with high primary stability (Fig. 7). Standard implant bridge abutments (AMCZ abutments, Southern Implants, South Africa) were then torqued into place onto all four zygomatic implants with longer 5 mm versions being used on the defect side to facilitate the later flap perforation. The soft tissues of the right maxilla were then closed with multiple resorbable sutures.

**Fig. 8:**
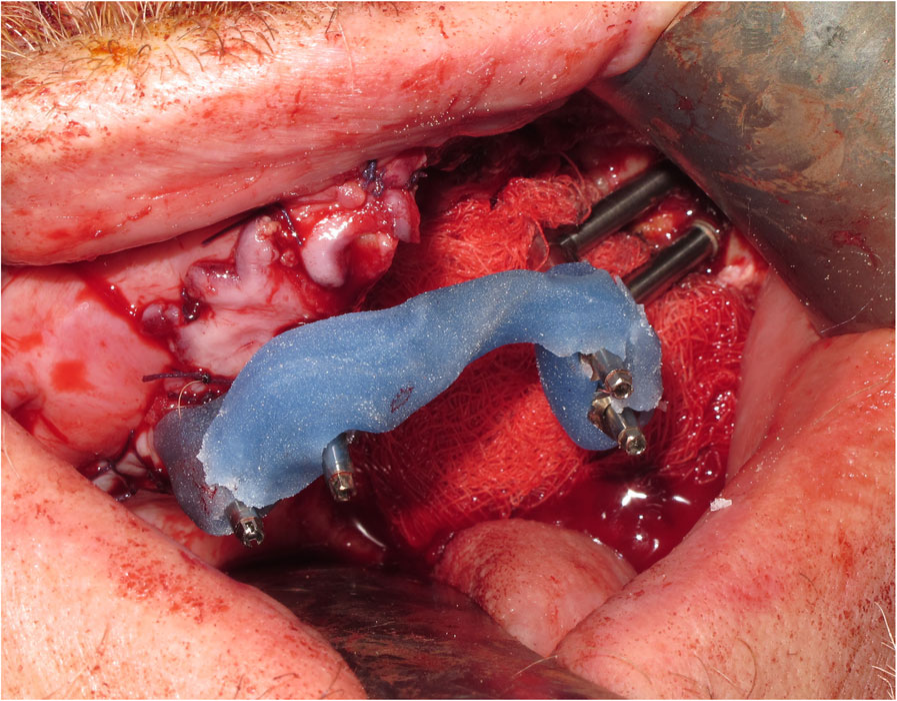
Abutment level impression utilising light-cured acrylic tray material

**Fig. 9:**
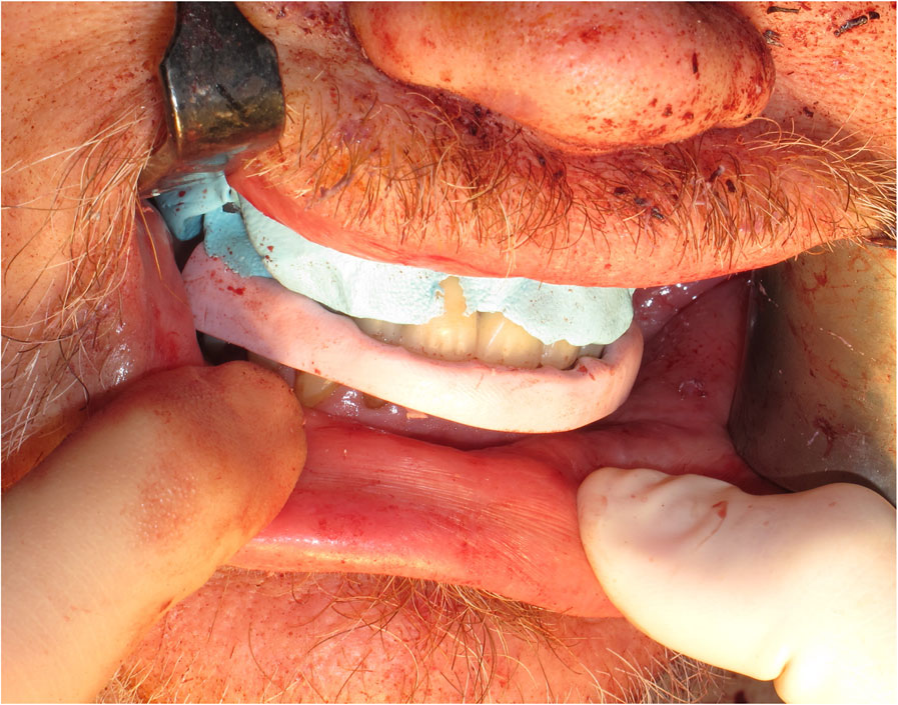
Inter-occlusal registration using the pre-fabricated maxillary denture prosthesis relined with silicone putty over the implant abutment protection caps

The implant positions were then accurately registered by utilising light-cured resin tray material (Individo® Lux, Voco Gmbh, Germany) and abutment level impression copings. The resin material was applied in sections around the impression copings and cured incrementally to ensure a rigid splinting of the impression copings (Fig. 8). Abutment protection caps were then placed over all four abutments prior to the jaw registration procedure which was undertaken using the pre-fabricated denture appliance relined with silicone putty material (Provil soft putty, Heraeus Kulzer GmbH) (Fig. 9).

**Fig. 10:**
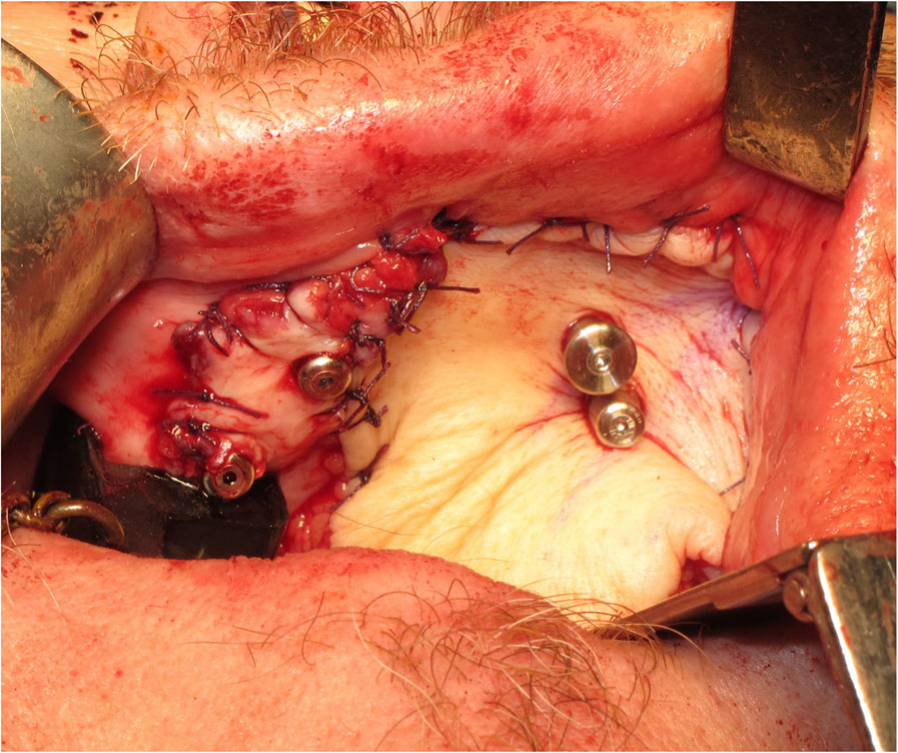
Radial forearm flap inset and sutured into the maxillary defect and perforated by the zygomatic oncology implant abutments

The radial forearm free flap (RFFF) was then disconnected from the arm and inset into the maxillary defect after creating a tunnel down into the left neck for the pedicle. The flap was carefully perforated over the zygomatic implant abutment protection caps using a short incision just through the skin layer followed by blunt dissection to allow the abutment and cap to perforate the flap ensuring a tight adaptation of the flap around the abutment (Fig. 10). The flap anastomosis was then completed utilising the operating microscope and the neck and arm wounds closed. The patient recovered well from the surgery and was subsequently discharged at 8 days post-operatively. The tumour and neck dissection specimens were examined and reported as pT4a NO M0 squamous cell carcinoma of the left maxilla with a 7.2 mm depth of invasion. There was a close anterior mucosal margin of 1.3 mm and the decision was therefore taken for post-operative adjuvant radiotherapy.

**Fig. 11:**
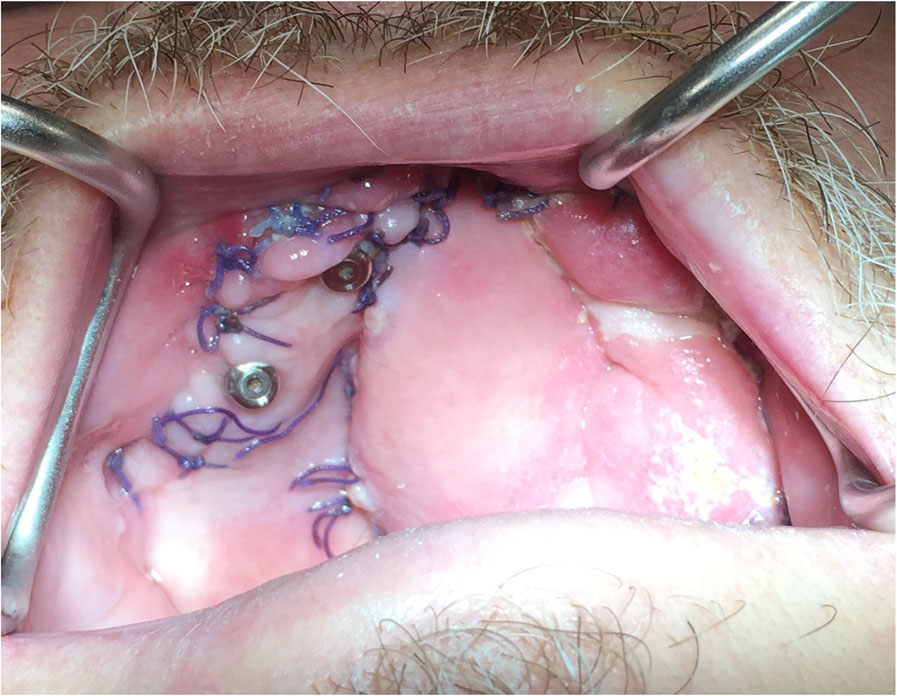
Intra-oral view of the soft tissue flap at 3 weeks post-operatively with overgrowth of flap over the zygomatic oncology implants

**Fig. 12:**
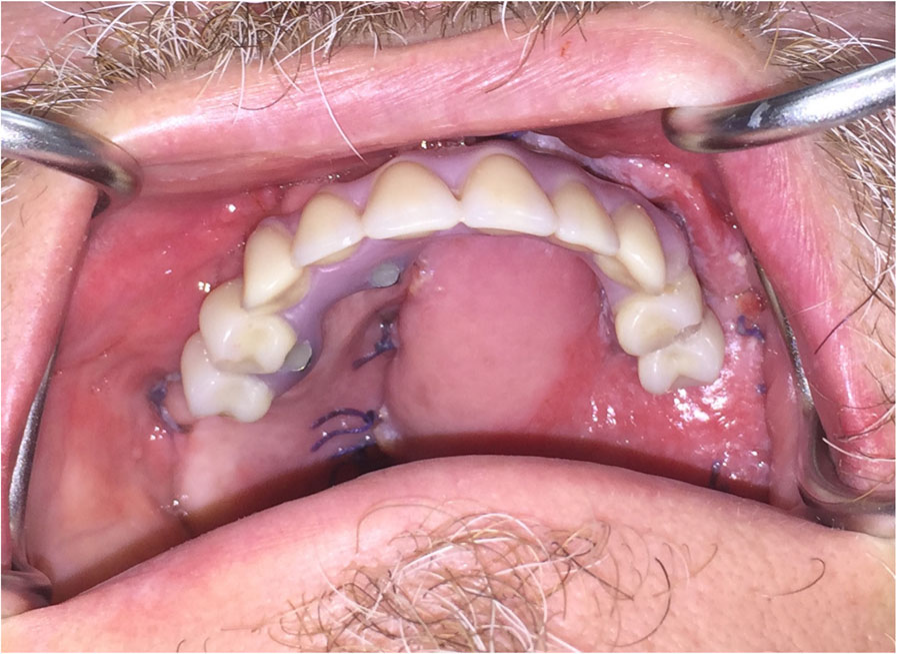
Provisional acrylic fixed dental prosthesis fitted at 4 weeks post-surgery

**Fig. 13:**
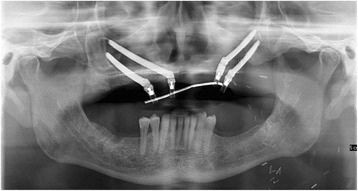
Panoramic dental radiograph showing the position of the zygomatic implants and the seating of the initial fixed prosthesis

**Fig. 14:**
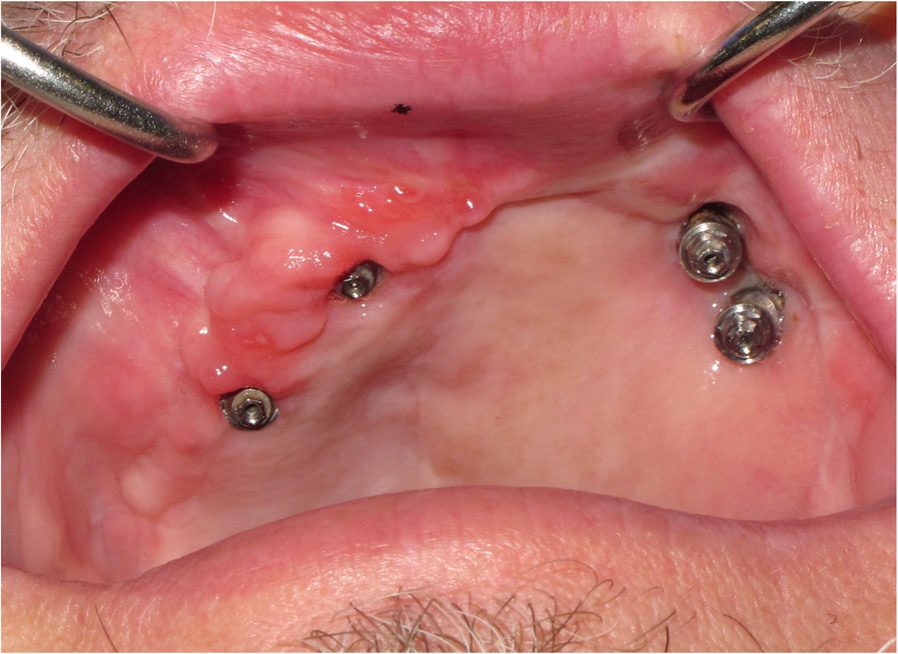
Intra-oral view of perforated flap 3 weeks following radiotherapy

**Fig. 15:**
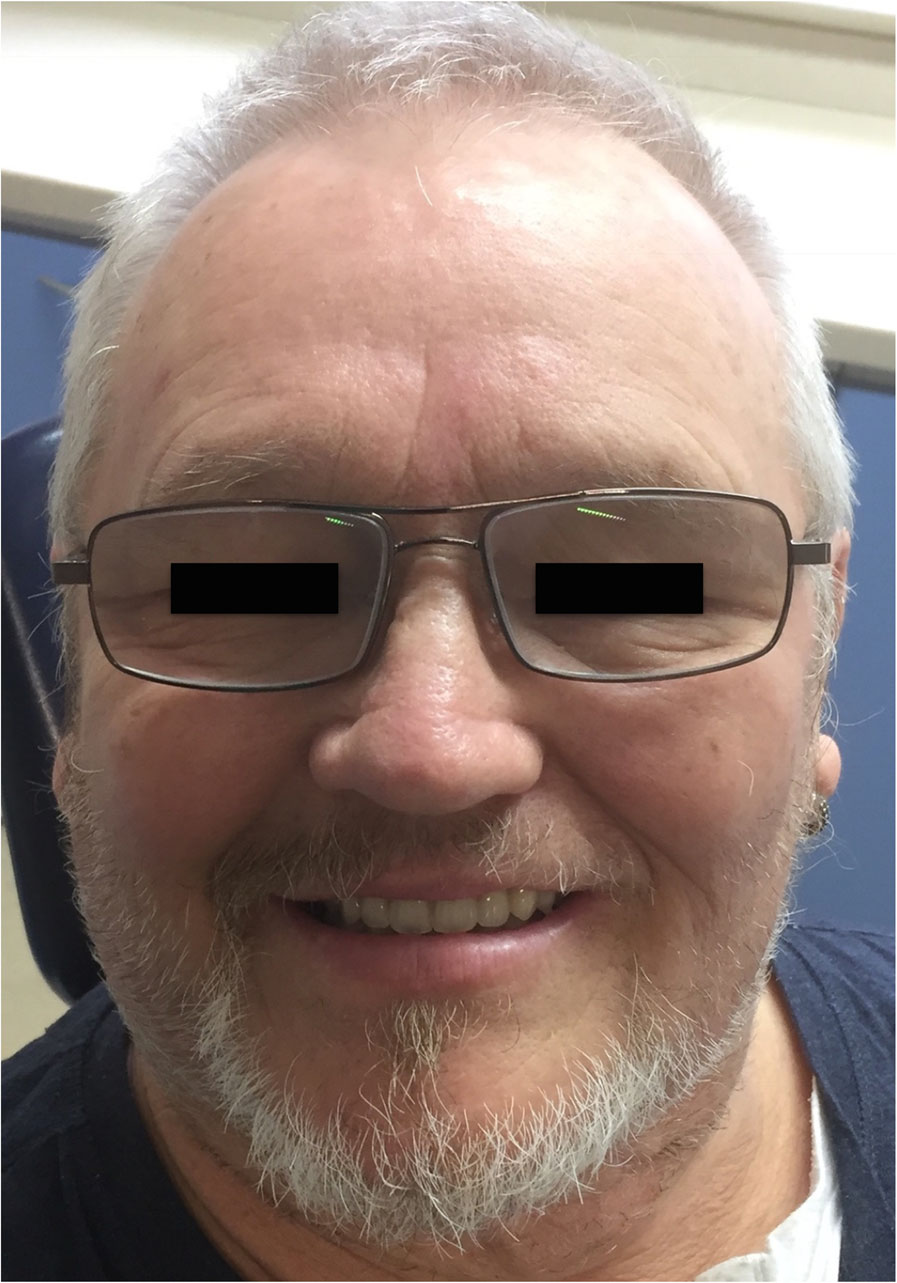
Facial appearance 18 months following treatment

Three weeks post-surgery, the patient was seen for review and to try-in the provisional prosthesis. Unfortunately, in the interim, the RFFF had overgrown the zygomatic implants (Fig. 11.) and so, under local anaesthesia, the implants were re-exposed to allow the provisional prosthesis to be tried in. The incisal level of the prosthesis was modified, and the prosthesis was then finalised in the laboratory and fitted 1 week later, 1 month following surgery (Fig. 12). A post-fitting radiograph demonstrated good positioning of the implants and seating of the initial prosthesis (Fig. 13). The patient then completed 6 weeks of radiotherapy (63 Gy in 30 fractions). He subsequently attended with a fracture of the provisional prosthesis 3 weeks after completion of radiotherapy when the bridge was removed for repair. All implants were firmly integrated, the initial oral ulceration was now settling and the flap reconstruction was performing well with no evidence of breakdown or dehiscence (Fig. 14). The bridge was repaired and re-fitted the same day, and arrangements were made for the construction of a new definitive acrylic bridge with a cobalt-chrome framework which was subsequently fitted for the patient. The patient continued to be followed up, and 12 months following surgery completed a quality of life feedback questionnaire [7] where he rated his overall quality of life as “very good” and scored maximally in most domains with the exception of speech and fear of recurrence (Table 1). At 18 months post-surgery, the patient was still disease free with no further incidents of prosthodontic related complications since the definitive bridge was fitted. His facial appearance (Fig. 15) was symmetrical with no significant distortion despite his previous maxillary resective surgery.

**Table 1:** Patient-reported quality of life outcomes following ZIP flap procedure

Domain	Score
Activity	100 (“I am as active as I have ever been”)
Anxiety	100 (“I am not anxious about my cancer”)
Appearance	75 (“The change in my appearance is minor”)
Chewing	100 (“I can chew as well as ever”)
Fear	75 (“I have a little fear, with occasional thoughts but they don’t really bother me”)
Intimacy	100 (“I have no problems with intimacy as a result of my cancer”)
Mood	100 (“My mood is excellent and unaffected by my cancer”)
Pain	100 (“I have no pain”)
Recreation	100 (“There are no limitations to recreation at home or away from home”)
Saliva	100 (“My saliva is of normal consistency”)
Speech	75 (“I have difficulty saying some words but I can be understood over the phone”)
Shoulder	100 (“I have no problem with my shoulder”)
Swallowing	100 (“I can swallow as well as ever”)
Taste	100 (“I can taste food normally”)
Overall QOL	Very good
Most important aspect	Fear of recurrence

### Procedural modifications to the ZIP flap technique

**Fig. 16:**
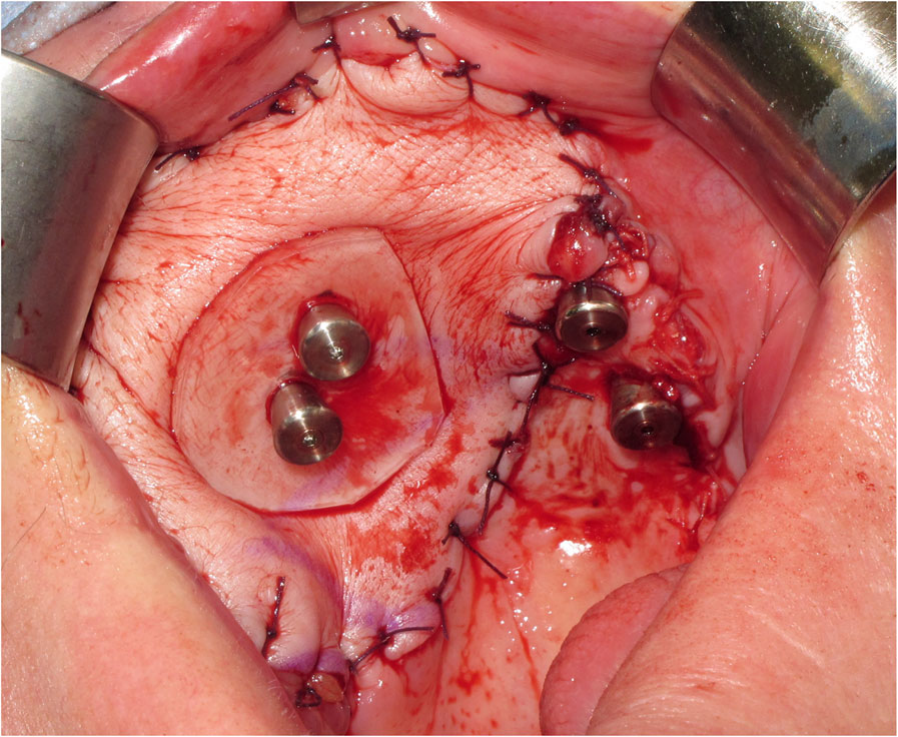
Another ZIP flap case demonstrating the use of a perforated polythene “washer” to keep the flap from overgrowing the implant abutments during the healing phase

**Fig. 17:**
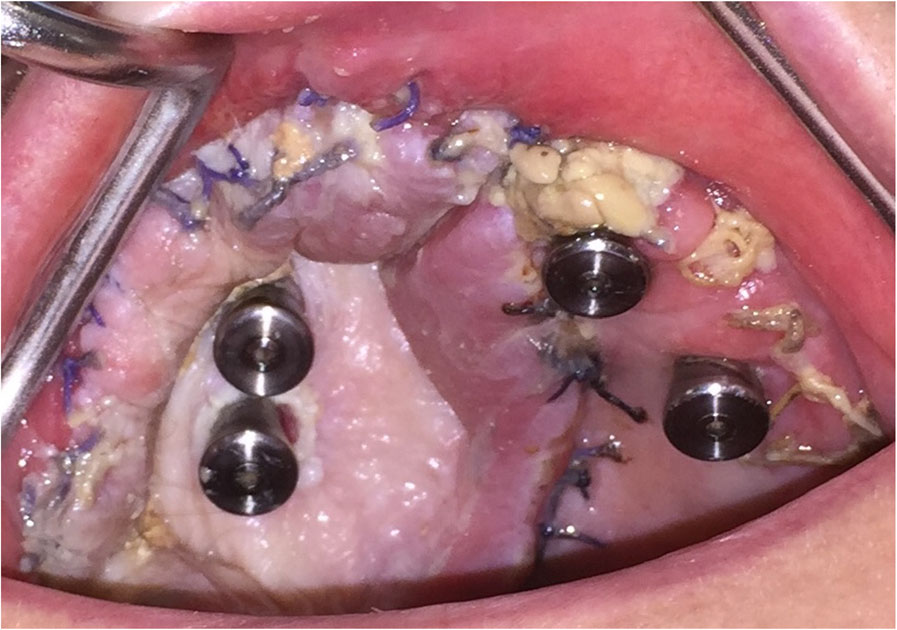
The appearance of the case shown in Fig. 16 with the polythene “washer” removed at 2 weeks post-surgery, providing access to the zygomatic oncology implants

In order to address some of the issues highlighted in this early case, the technique was modified slightly to try and prevent flap overgrowth and prosthesis fracture in the early stages. In order to prevent flap overgrowth over the zygomatic oncology implant abutments, the use of a polythene washer was instituted on subsequent cases treated in the unit. Once the flap was perforated, a 2-mm thick polythene sheet (Centriform Soft Mouthguard material, WHW Plastics Ltd., Hull, UK) was taken and a small disc cut out corresponding to an area of 1–2 cm^2^ surrounding the zygomatic oncology implants. Using a 5-mm tissue biopsy punch, holes were cut into the sheet corresponding to the positions of the abutments and the perforated polythene sheet was then placed over the abutments to keep the flap in a superior position during the initial healing phase prior to restoration. The polythene washer was then kept in place using conical abutment protection caps (Fig. 16), and this enabled the prevention of flap tissue overgrowth and retained access to the oncology implants for subsequent restoration (Fig. 17). In view of the fracture of the interim prosthesis reported in this case, the technique was modified with a definitive cobalt chrome framework being constructed within the first 2 weeks post-surgery with one visit for try-in of the framework and tooth set-up being scheduled to allow any modifications required to either incisal level, occlusion and overall soft tissue fit to be completed. This try in visit occurred at 2–3 weeks post-surgery with the final fit occurring 1 week later. This has prevented further issues for all subsequent patients.

### Discussion

In order to reduce intra-operative time, the soft tissue free flap is harvested at the same time as the implant placement and prosthodontic procedures. On raising a skin island, it is appropriate to make it a little over-sized for the required defect to ensure that tension and possible dehiscence at the surgical margins during healing is reduced.

In low-level maxillectomy (Brown class II), the need for bony reconstruction is questionable depending on the horizontal component. With the preservation of the orbital floor, zygomatic prominence and some bony support for the nose, facial appearance, in the experience of the authors and, as demonstrated by this case, is not significantly worsened despite low-level removal of the maxilla. The key issues in these low level defects are adequate clearance of tumour, dealing with the oro-nasal communication and reconstruction of the dentition. Whilst prosthodontic obturation can deal with these aspects in a simple manner, the stability of the obturator prosthesis and its ability to completely seal the oro-nasal defect has limitations. In addition, these prostheses require a significant amount of adjustments, clinic visits and on-going maintenance. The soft lining materials perish, discolour and harbour surface biofilm often resulting in some mal-odour and the need for regular replacement. For many patients, there is a psychological impact of retaining the maxillectomy defect and high anxiety related to the insertion and removal of the prosthesis as well as concerns relating to the handicap they would experience to speech, and eating should their prosthesis fracture or fail in some way. The use of implants to retain maxillary obturators certainly improves their stability and retention, but efficacy of the oro-nasal seal still requires regular maintenance and patients still often dislike the hygiene aspects of looking after the defect and their implant supra-structure within the defect.

The use of soft tissue flaps to close a typical hemi-maxillectomy defect is an effective way of dealing with the oro-nasal communication, but in isolation, this technique works against dental rehabilitation as the bulk of the flap provides a very poor moveable foundation for a subsequent removable prosthesis. The move towards the use of composite reconstruction (especially the fibula flap) has been facilitated by the use of digital planning in which dental implants can be inserted into the fibula flap at the time of harvest and inset facilitated by the use of stereolithographic guides. However, this procedure is not widely applicable for all patients due to financial, technological and medical restrictions and is not currently able to provide patients with an early loaded fixed dental prosthesis especially when post-operative radiotherapy is being utilised. Many older patients presenting with maxillary malignant tumours also have significant peripheral vascular disease and other significant medical co-morbidities which may prevent the harvest of a vascularised composite flap.

In contrast, the use of a soft tissue flap such as the RFFF or antero-lateral thigh flap can often be safely employed in elderly patients with peripheral vascular disease without unduly lengthening the operation too significantly with two-team operating. In addition, the predictability of these flaps with their excellent pedicle lengths is ideal for closure of the resulting oro-nasal surgical defect. The use of a slightly oversized graft is recommended to ensure that any tension on the wound peripheries is kept to a minimum during the healing phase. In addition, for those patients undergoing post-operative radiotherapy, a degree of shrinkage and tightening of the flap tissues is to be expected.

Immediate/early loading of zygomatic [8] and dental implants [9] have been well demonstrated already within the literature with very high implant survival rates. In the oncology setting, Boyes-Varley et al. [4] lost no zygomatic/oncology implants in their series of 20 patients restored with implant-retained obturators, 6 of whom received radiotherapy post-operatively. The case reported has been followed up for 18 months so far without evidence of zygomatic implant failure despite the use of radiotherapy. A recent review of conventional zygomatic implant surgery demonstrated that the incidence of failure after the 6-month stage was extremely low [8] although for zygomatic oncology implants, this data is not yet fully reported in the literature with the only data available on zygomatic oncology implants being limited to the work of Boyes-Valey [4], Pellegrino [10] and the authors themselves [6]. The removal of teeth at primary cancer surgery to facilitate placement of implants on the non-defect side requires careful consideration; where teeth are of poor prognosis with poor bone support, it is easier to extract, perform localised osteoplasty prior to the insertion of a conventional zygomatic implant with its inherent excellent stability and ability to be loaded early in the post-operative period. Where teeth have excellent bone support but additional implants are required to facilitate the construction of a fixed prosthesis, then careful extraction of selected teeth with the immediate installation of a root form implant can be utilised with good success as long as high primary stability is achieved at these sites.

Whilst technically, it would be possible to construct and fit the prosthesis on the same day or even a week later, the need for microvascular flap monitoring in the immediate post-operative period, together with the significant recovery period required by the patient following surgery has lead the authors to delay the fitting of the prosthesis at the 4 to 6-week period post-operatively. In terms of ongoing clinical implant follow-up, no attempt was made at peri-implant probing for the oncology zygomatic implants perforating the soft-tissue flap as it was deemed important not to disturb the soft tissue seal of the skin flap around the implant abutments. No discharge or suppuration was noted during follow-up in this case. Periodontal probing around the conventionally placed zygomatic implants was undertaken periodically during follow up and remained within normal limits.

The use of a soft tissue rather than composite reconstruction may also facilitate a shorter hospital stay and allow adjuvant radiotherapy to be delivered in a more rapid timescale with possible impact on overall cure rates of this very debilitating tumour. The initial experiences with this procedure in over ten cases have been extremely positive with excellent appreciation by patients who value being provided with a fixed dental prosthesis so quickly after major surgery.

## Conclusions

The ZIP flap technique represents an innovative approach to the management of patients presenting with low-level malignant maxillary tumours. It provides effective closure of the resulting maxillary defect restoring speech and swallowing functions and also establishing a high-quality fixed dental rehabilitation in a rapid timescale, thus facilitating a more timely return to function and restored facial appearance. This approach has now been adopted routinely in the unit and it is hoped that a cases series will be presented in due course with more detailed patient outcomes. Further work on the long-term results of the ZIP flap procedure is required together with an ongoing appreciation of the important case selection factors for this treatment protocol.
